# Functional characterization and key residues engineering of a regiopromiscuity *O*-methyltransferase involved in benzylisoquinoline alkaloid biosynthesis in *Nelumbo nucifera*

**DOI:** 10.1093/hr/uhac276

**Published:** 2022-12-09

**Authors:** Yuetong Yu, Yan Liu, Gangqiang Dong, JinZhu Jiang, Liang Leng, XianJu Liu, Jun Zhang, An Liu, Sha Chen

**Affiliations:** Key Laboratory of Beijing for Identification and Safety Evaluation of Chinese Medicine, Institute of Chinese Materia Medica, China Academy of Chinese Medical Sciences, No. 16, Nanxiaojie, Dongzhimennei, Beijing 100700, China; Key Laboratory of Beijing for Identification and Safety Evaluation of Chinese Medicine, Institute of Chinese Materia Medica, China Academy of Chinese Medical Sciences, No. 16, Nanxiaojie, Dongzhimennei, Beijing 100700, China; Amway (China) Botanical R&D Centre, Wuxi 214115, China; Key Laboratory of Beijing for Identification and Safety Evaluation of Chinese Medicine, Institute of Chinese Materia Medica, China Academy of Chinese Medical Sciences, No. 16, Nanxiaojie, Dongzhimennei, Beijing 100700, China; Key Laboratory of Beijing for Identification and Safety Evaluation of Chinese Medicine, Institute of Chinese Materia Medica, China Academy of Chinese Medical Sciences, No. 16, Nanxiaojie, Dongzhimennei, Beijing 100700, China; Key Laboratory of Beijing for Identification and Safety Evaluation of Chinese Medicine, Institute of Chinese Materia Medica, China Academy of Chinese Medical Sciences, No. 16, Nanxiaojie, Dongzhimennei, Beijing 100700, China; Key Laboratory of Beijing for Identification and Safety Evaluation of Chinese Medicine, Institute of Chinese Materia Medica, China Academy of Chinese Medical Sciences, No. 16, Nanxiaojie, Dongzhimennei, Beijing 100700, China; Key Laboratory of Beijing for Identification and Safety Evaluation of Chinese Medicine, Institute of Chinese Materia Medica, China Academy of Chinese Medical Sciences, No. 16, Nanxiaojie, Dongzhimennei, Beijing 100700, China; Key Laboratory of Beijing for Identification and Safety Evaluation of Chinese Medicine, Institute of Chinese Materia Medica, China Academy of Chinese Medical Sciences, No. 16, Nanxiaojie, Dongzhimennei, Beijing 100700, China

## Abstract

Lotus (*Nelumbo nucifera*), an ancient aquatic plant, possesses a unique pharmacological activity that is primarily contributed by benzylisoquinoline alkaloids (BIAs). However, only few genes and enzymes involved in BIA biosynthesis in *N. nucifera* have been isolated and characterized. In the present study we identified the regiopromiscuity of an *O*-methyltransferase, designated NnOMT6, isolated from *N. nucifera*; NnOMT6 was found to catalyze the methylation of monobenzylisoquinoline 6-*O*/7-*O*, aporphine skeleton 6-*O*, phenylpropanoid 3-*O*, and protoberberine 2-*O*. We further probed the key residues affecting NnOMT6 activity via molecular docking and molecular dynamics simulation. Verification using site-directed mutagenesis revealed that residues D316, N130, L135, N176A, D269, and E328 were critical for BIA *O*-methyltransferase activities; furthermore, N323A, a mutant of NnOMT6, demonstrated a substantial increase in catalytic efficiency for BIAs and a broader acceptor scope compared with wild-type NnOMT6. To the best of our knowledge, this is the first study to report the *O*-methyltransferase activity of an aporphine skeleton without benzyl moiety substitutions in *N. nucifera*. The study findings provide biocatalysts for the semisynthesis of related medical compounds and give insights into protein engineering to strengthen *O*-methyltransferase activity in plants.

## Introduction

Benzylisoquinoline alkaloids (BIAs) are structurally diverse natural products from lotus (*Nelumbo nucifera*) and plants belonging to the orders Ranunculales and Magnoliales; they possess important bioactivities and pharmacological values [1, 2]. Several BIA compounds are drugs, including the analgesics morphine and codeine from *Papaver somniferum* and the antibacterial berberine from *Coptis japonica* [3, 4]. The complicated stereoscopic configurations of BIAs render it difficult to modify their structure using chemical synthesis. Until now, these plants have remained the most economical source of these drugs. The medical development of BIAs is limited by the supply and demand conflict [5]. Therefore, there is an urgent need to develop commercially feasible engineering for BIA biosynthesis based on the discovery of BIA biosynthetic genes with biocatalytic activities.

Methylation is crucial to the structural and functional variety of BIAs. The chemical properties of BIAs can be changed by adding methyl groups, such as steric effects, overall hydrophobicity, and electronic properties, thereby shifting their biological activity [6]. In plants, *O*-methylation, a typical modification of BIA metabolism, is catalyzed by *O*-methyltransferases (OMTs), offering a highly efficient and regio/stereospecific approach to the methylation of acceptors. Dozens of OMTs involved in the BIA biosynthetic pathway have been isolated from several species in the order Ranunculales [6]*.* Recently, seven OMTs from *Corydalis yanhusuo* and two OMTs from *Liriodendron* belonging to the order Magnoliales were functionally characterized [7, 8]. These OMTs primarily catalyzed the methylation of three BIA scaffolds: 1-benzylisoquinolines, protoberberine, and phthalideisoquinolines, including norcoclaurine 6-*O*-methyltransferase (6OMT), 3′-hydroxy-*N*-methylcoclaurine 4′-*O*-methyltransferase (4′OMT), reticuline 7-O-methyltransferase (7OMT), norreticuline 7-*O*-methyltransferase (N7OMT), scoulerine-9-*O*-methyltransferase (SOMT), scoulerine 2-*O*-methyltransferase (S2OMT), columbamine *O*-methyltransferase (CoOMT), and 4′-*O*-desmethyl-3-*O*-acetylpapaveroxine 4′-*O*-methyltransferase (PsOMT2:PsOMT3 and PsOMT2:Ps6OMT heterodimers) [9–17]. The identification of more OMTs that catalyze diverse BIA skeletons and at different positions contributes to the elucidation of the metabolic pathways of BIAs and their application as biocatalysts in synthetic biology platforms.

The crystal structures of two OMTs, Tf6OMT (PDB:5ICE) and PsSOMT (PDB:6I6K), have been reported; moreover, these studies provide a molecular-level understanding of the reaction mechanisms of OMTs [18, 19]. However, few studies have reported the protein engineering of these OMTs. To understand the catalytic mechanism and rational design of these OMTs for the purpose of protein engineering, it is important to obtain knowledge about their protein function by probing key amino acid residues in order to improve their activity, alter their regioselectivity, or expand their substrate scope.

**Figure 1:**
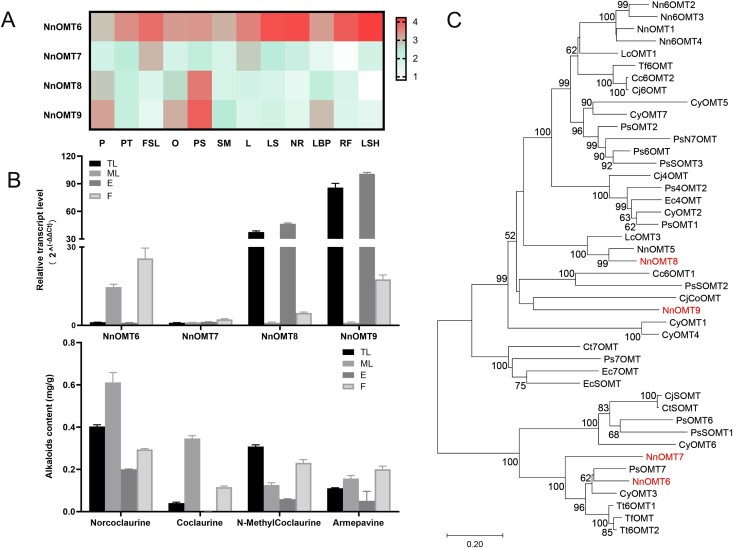
Isolation of NnOMTs and phylogenetic tree. (A) Expression heat map of NnOMTs in 12 organs of *N. nucifera*. Expression values are shown as exponential FPKM values with base 10. (B) Relative expression levels and alkaloid contents of NnOMTs in tender leaf (TL), mature leaf (ML), flower (F), and embryo (E). (C) Phylogenetic tree of NnOMT candidates and previously characterized OMTs in BIA biosynthesis.

The lotus, which belongs to the order Proteales, is an aquatic plant known across Asia for its nutritional and therapeutic benefits [20]. According to research, BIAs are among its primary bioactive components, and include three skeletons of monobenzylisoquinolines, aporphine, and bisbenzylisoquinolines [21]. For example, the two monobenzylisoquinoline alkaloids norcoclaurine and coclaurine exhibit anti-HIV activity [22]. Armepavine is a potential therapeutic treatment for autoimmune illnesses, such as autoimmune crescentic glomerulonephritis and systemic lupus erythematosus [23, 24]. The aporphine alkaloid nuciferine exhibits antidiabetic, anti-HIV, antimelanogenesis, and anticancer properties [25–27]. Neferine, a bisbenzylisoquinoline, exerts antiarrhythmic effects and shows antitumor activities for the lungs, liver, and breast [28–31]. However, little is known regarding the molecular mechanisms underlying the biosynthesis of BIAs in lotus. Until now, only two OMTs involved in BIA metabolism in lotus have been functionally characterized; they catalyze the 6-*O*- and 7-*O*-methylation of the 1-benzylisoquinoline backbone, respectively [32]. In lotus, the 1-benzylisoquinoline backbone was mainly *O*-methylated at the C6, C7, and/or C4′ positions to yield a variety of 1-benzylisoquinoline alkaloid derivatives [1]. Aporphine and bisbenzylisoquinoline alkaloids in lotus are derived from monobenzylisoquinoline; however, there are no reports of OMTs catalyzing the aporphine and bisbenzylisoquinoline backbone in lotus. In the present study we report a novel and regiospecific *O*-methyltransferase, designated NnOMT6, which was involved in BIA biosynthesis in *N. nucifera*. We demonstrate that NnOMT6 catalyzes the methylation of phenylpropanoid, 1-benzylisoquinoline, aporphine, and protoberberine skeletons. Furthermore, we partly elucidated the catalytic mechanisms of NnOMT6, engineered key residues for *O*-methylation based on a semi-rational design, and obtained a mutant with stronger activity and wider acceptor scope.

## Results

### Isolation of OMT candidates from *N. nucifera*

Four putative NnOMT genes were screened through a BLASTP search of the transcriptome data of *N. nucifera* using functionally characterized BIA OMTs as homology-based templates (sequence information of NnOMTs is shown in Supplementary Data Table S1). Because five OMTs (NnOMT1–5) have been functionally characterized in a previous report [32], the full-length cDNAs of the new NnOMT candidates in our study were designated NnOMT6 (Gene ID: 104585771), NnOMT7 (Gene ID: 104601399), NnOMT8 (Gene ID: 109115720), and NnOMT9 (Gene ID: 109115720). NnOMT6–NnOMT9 contain open reading frames of 1095, 1101, 1047, and 1101 bp encoding 364, 366, 348, and 366 amino acids, with corresponding theoretical molecular weights of 39.7, 40.5, 38.2, and 40.5 kDa, respectively.

**Figure 2:**
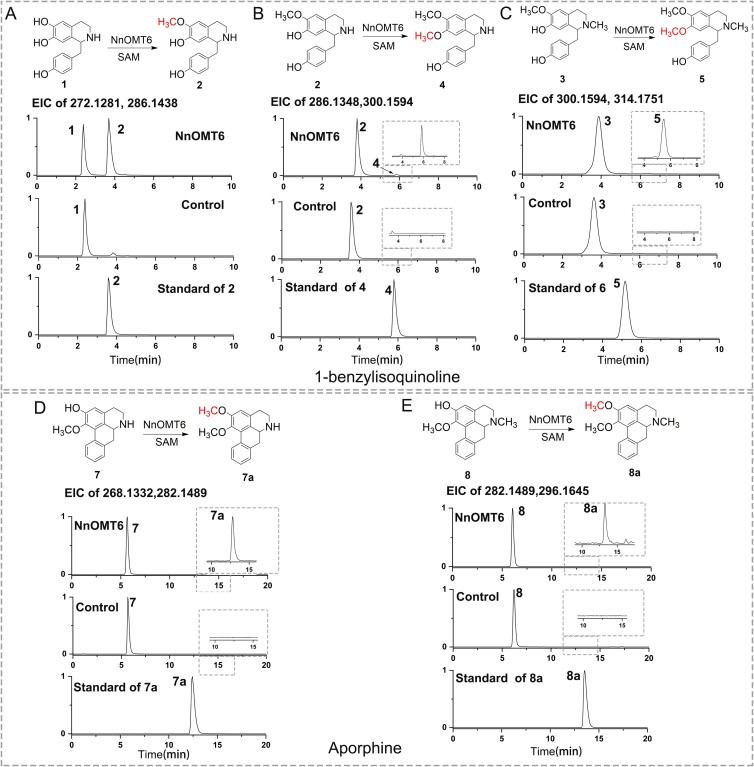
*O*-Methylation of compounds **1**, **2**, **3**, **7**, and **8** catalyzed by NnOMT6. (A) Reaction and extracted ion chromatograms (EICs) of *m*/*z* 272.1281 and 286.1438 with (*S*)-norcoclaurine (**1**) as the substrate. (B) Reaction and EICs of *m*/*z* 286.1438 and 300.1594 with (*S*)-coclaurine (**2**) as the substrate. (C) Reaction and EICs of *m*/*z* 300.1594 and 314.1751 with *N*-methylcoclaurine (**3**) as the substrate. (D) Reaction and EICs of *m*/*z* 268.1332 and 282.1489 with asimilobine (**7**) as the substrate. (E) Reaction and EICs of *m*/*z* 282.1489 and 296.1645 with *N*-methylasimilobine (**8**) as the substrate.

The transcriptome databases of different parts of *N. nucifera*, including its leaf stalk (LS), flower stalk (FS), new root (NR), leaf bud primordium (LBP), root fibril (RF), leaf sheath (LSH), leaf (L), pistil (P), petal (PT), flower sepal (FSL), ovary (O), petalized stamen (PS), and stamen (SM), were constructed using Illumina paired-end sequencing technology. The expression of the four candidate NnOMTs in different parts of *N. nucifera* was investigated based on the transcriptome databases. FPKM (fragments per kilobase of transcript per million mapped reads) values showed that NnOMT6 expression was remarkably higher in the leaf tissues (LS and LSH) than in the flower tissues (P, PT, FSL, O, PS, and SM) (Fig. 1A). NnOMT7 showed higher expression levels in FSL and L than in other tissues. The expression levels of NnOMT8 and NnOMT9 were higher in PS than in L, LS, and LSH.

To explore the relationship between NnOMTs and correlated BIAs, we investigated their expression patterns in four tissues [tender leaf (TL), mature leaf (ML), flower (F), and embryo (E)], and the amounts of norcoclaurine, coclaurine, *N*-methylcoclaurine, and armepavine were determined. NnOMT6 expression levels were markedly higher in ML and F than in TL and E. Conversely, NnOMT8 and NnOMT9 showed high expression levels in TL and E (Fig. 1B). The NnOMT7 expression level was relatively consistent in all parts of lotus, and it was more abundant in F. The highest accumulation of coclaurine (the product of 6-OH methylation of norcoclaurine) was in ML, F, and TL, whereas lower levels were detected in E. The amount of *N*-methylcoclaurine was highest in TL, followed by F, ML, and E. The accumulation of armepavine was relatively higher in ML and F. The expression patterns of NnOMT6 were consistent with the accumulation of coclaurine in four tissues. Therefore, NnOMT6 was selected as a possible functional gene involved in coclaurine synthesis.

### Phylogeny of NnOMT candidates

We used MEGA 7.0 software to conduct phylogenetic analysis for the four candidate genes, together with known BIA OMTs from the orders Ranunculales, Magnoliales, and Proteales. The results revealed that the NnOMTs (Nn6OMT2, Nn6OMT3, Nn6OMT4, NnOMT1) reported in the literature were clustered together. In our study, NnOMT6 and NnOMT7 were clustered with CyOMT3 and three known OMTs from *Thalictrum tuberosum* [33] (Fig. 1C). NnOMT6 shared 75.21%, 73.96% and 74.09% amino acid sequence identity with TtOMT, Tt6OMT1, and Tt6OMT2, respectively; these three OMTs modify phenylpropanoid and several other BIA skeletons in *T. tuberosum*. Similarly, NnOMT7 showed a high sequence identity (>60%) with TtOMT, Tt6OMT1, and Tt6OMT2. NnOMT8 was grouped into a clade with NnOMT5 with 84.59% amino acid sequence identity, and NnOMT9 clustered with CjCoOMT with 41.28% identity.

### Molecular cloning and functional characterization of NnOMTs

The four candidate genes (NnOMT6–9) were cloned into a pET28a(+) vector, then expressed in *Escherichia coli* BL21 (DE3) strains. To characterize the catalytic activity of putative NnOMTs *in vitro*, 12 potential BIA substrates (Supplementary Data Fig. S2), including 6 monobenzylisoquinoline (**1–6**), 3 aporphine (**7–9**), and 3 bisbenzylisoquinoline (**10–12**) alkaloids, were used as substrates. *S*-Adenosyl-l-methionine (SAM) was used as the methyl donor. The initial reaction was tested using crude protein and analyzed using ultraperformance liquid chromatography–electrospray ionization–quadrupole time of flight tandem mass spectrometry (UPLC–ESI–QToF–MS/MS). Heat-inactivated enzymes (100°C, 10 minutes) were used as negative controls. Results showed that only NnOMT6 exhibited differential OMT catalytic activity for **1**, **2**, **3**, **7**, and **8** (Fig. 2), and the catalytic activity was confirmed with purified NnOMT6 (Supplementary Data Fig. S1). UPLC–MS analysis showed that NnOMT6 effectively catalyzed the 6-*O*-methylation of (*S*)-norcoclaurine (**1**) to yield (*S*)-coclaurine (**2**), which was identified by comparing the products with a reference standard (Fig. 2A). NnOMT6 also catalyzed the 7-*O*-methylation of (*S*)-coclaurine (**2**), and the product was identified as (*S*)-norarmepavine (**4**) based on a reference standard (Fig. 2B). NnOMT6 showed weak catalytic activity (<2%) when **3**, **8**, and **9** were used as substrates. Product peaks **5**, **7a**, and **8a** were detected and identified as armepavine, *N*-nornuciferine, and nuciferine by comparing them with the respective commercial standards (Fig. 2C–E). These results suggested that NnOMT6 can catalyze the 6-OH and 7-OH methylation of the monobenzylisoquinoline skeleton (**1**, **2**, **3**) without stereospecificity and only the 6-OH methylation of aporphine skeleton (**8**, **9**). No catalytic activities were found for liensinine (**10**), isoliensinine (**11**), and neferine (**12**), which are bisbenzylisoquinoline alkaloids.

### Further probe of substrate promiscuity of NnOMT6

**Figure 3:**
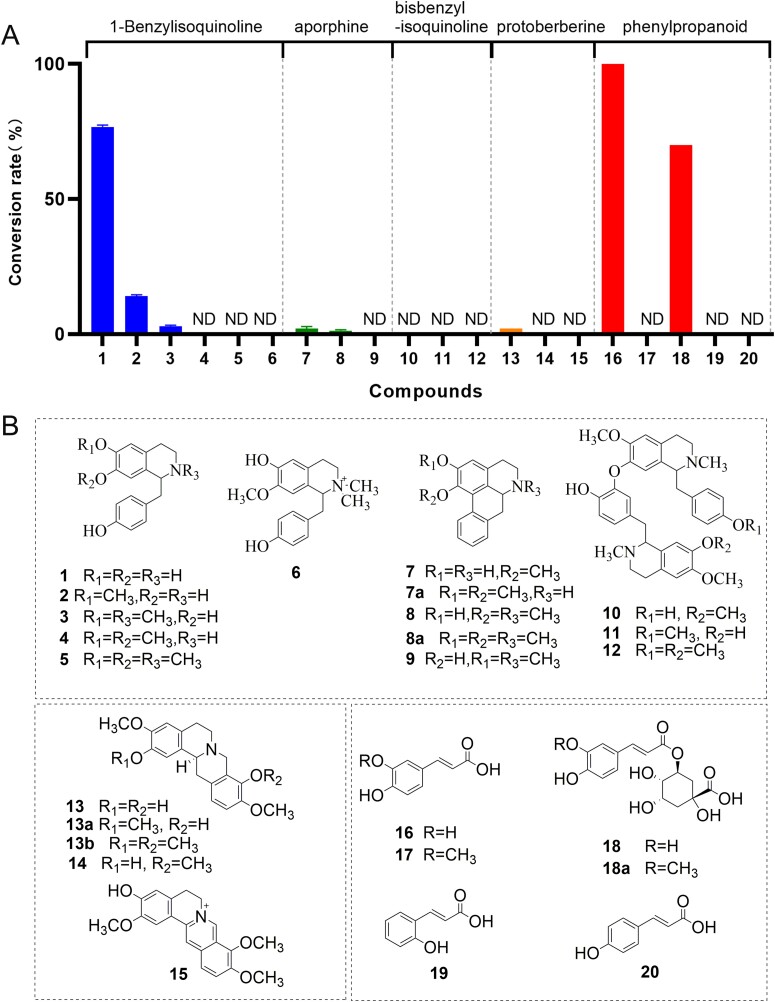
Exploring the catalytic promiscuity of NnOMT6. (A) Percent conversions of *O*-methylated products catalyzed by NnOMT6. (B) Structure of substrates **1**–**20** and corresponding *O*-methylated products. The substrate backbone contained mono-benzylisoquinoline, aporphine, bisbenzylisoquinoline, protoberberine, and phenylpropanoid.

To further explore the substrate promiscuity of NnOMT6, three protoberberine (**13**, **14**, **15**) and five phenylpropionic acid (**16**–**20**) substrates were used (Fig. 3). The results showed that NnOMT6 catalyzed the 3-*O*-methylation of caffeic acid (**16**), and all substrates were transformed to produce ferulic acid (**17**) (Supplementary Data Fig. S3). NnOMT6 displayed a higher turnover rate (74.1%) toward chlorogenic acid (**18**), producing three deprotonated ions at *m*/*z* 369.1180 and fragment ion at *m*/*z* 177.0537 (Supplementary Data Fig. S4). Compounds **18a**, **18b**, and **18c** were tentatively identified as 3-*O*-feruloylquinic acid, methyl-chlorogenate, and 4-*O*-feruloylquinic acid, respectively. A weak catalytic activity (<2%) was observed for (*S*)-scoulerine (**13**). The product **13a** (*m*/*z* 342.1700) was identified as (*S*)-tetrahydropalmatrubine with fragments at *m*/*z* 192.0998 and 165.0891, which were reported in a previous study [34] (Supplementary Data Fig. S5).

The catalytic efficiency of NnOMT6 was high for the phenylpropanoid compounds **16** (100%) and **18** (74.1%) and the 1-benzylisoquinoline compound **1** (76.7%); however, it was very low for **3**, **7**, **8**, and **13** (<2%). No catalytic ability was observed for **4**–**6**, **9**–**12**, **14**, **15**, **17**, **19**, and **20** (Fig. 3A). These results clearly indicated that NnOMT6 exhibited regiopromiscuity for several skeletons. NnOMT6 could catalyze the 6-*O-*/7-*O*-methylation of monobenzylisoquinoline, 6-*O*-methylation of aporphine, 2-*O*-methylation of protoberberine, and 3-*O*-methylation of phenylpropanoid skeletons.

### Biochemical characterization of recombinant NnOMT6 protein

The enzyme characteristics of NnOMT6 were studied (Supplementary Data Fig. S6). The pH and temperature conditions influencing enzyme activity were optimized. NnOMT6 exhibited its maximum activity at pH 8.0 (50 mM potassium phosphate) and 37°C. The changes in NnOMT6 products were linearly dependent on time (5–40 minutes).

The kinetic property of NnOMT6 was determined within a linear range of enzymatic reactions (Supplementary Data Fig. S7). The *K*_m_ values toward **1** and **16** were 281.73 and 216.03 μM, respectively. Further, the *K*_m_ value for SAM was 163.40 μM with caffeic acid (**16**) as a substrate. The *k*_cat_/K_m_ value (120.44 ± 13.10 M^−1^ s^−1^) with caffeic acid (**16**) was seven times higher than that with **1** (16.59 ± 1.48 M^−1^ s^−1^). These results indicated that caffeic acid was the preferred substrate of NnOMT6.

### Engineering key residues for the *O*-methyltransferase activity of NnOMT6

To further explore the catalytic mechanisms of the NnOMT6 enzyme, the key residues binding with *S*-adenosyl-l-homocysteine (SAH) and (*S*)-norcoclaurine were identified using molecular docking and molecular dynamics (MD) simulation.

**Figure 4:**
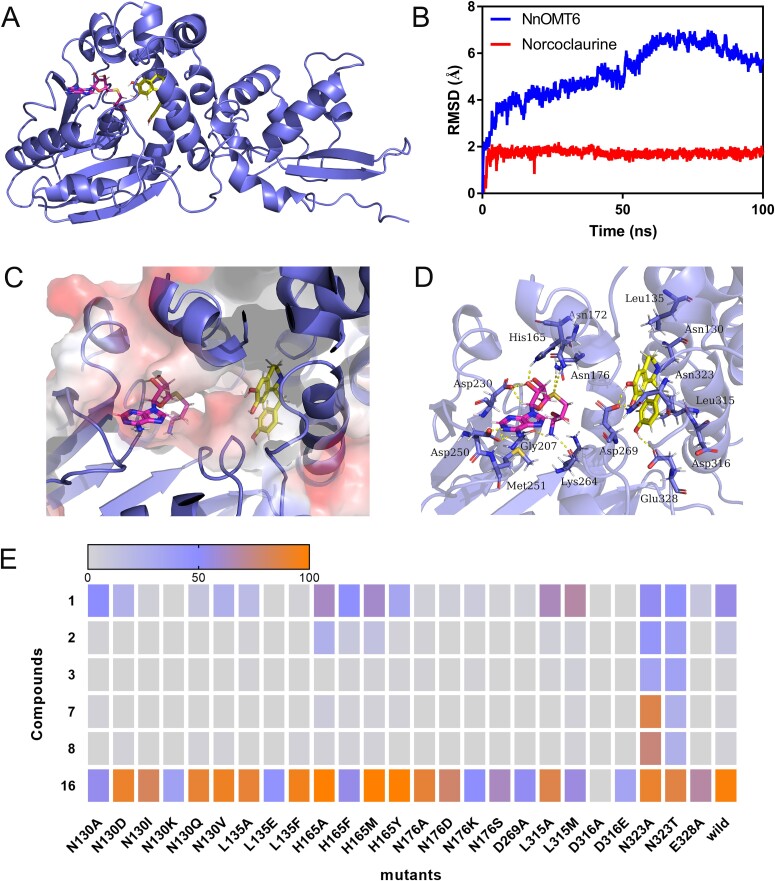
Semi-rational design and mutant screening for NnOMT6. (A) Binding model of the NnOMT6–SAH–(*S*)-norcoclaurine complex. (B) RMSD of NnOMT6 backbone and the substrate norcoclaurine. (C) Substrate binding pockets of NnOMT6 for (*S*)-norcoclaurine. (D) Amino acid residues surrounding SAH and norcoclaurine in the active site of NnOMT6. (*S*)-Norcoclaurine is colored yellow, SAH is colored magenta, and the residues surrounding the binding pocket are colored blue. Hydrogen bonds are depicted as yellow dashed lines. (E) Functional screening of mutants with compounds **1**, **2**, **3**, **7**, **8**, and **16** as substrates.

The homology structure of NnOMT6 was constructed with SWISS-MODEL using caffeic acid 3-*O*-methyltransferase (COMT; PDB ID: 1KYW) as the template. NnOMT6 shared 77.93% sequence identity with the template structure. The root mean square deviation (RMSD) value of the three-dimensional structure overlap between NnOMT6 and template is 0.095 Å, and both have the same alpha helix and beta strand regions.(Supplementary Data Fig. S8). The substrate (*S*)-norcoclaurine was docked into the structure of NnOMT6 using the Molecular Operating Environment (MOE) dock (Supplementary Data Fig. S9). Subsequently, all-atom MD simulations with explicit water were performed using AMBER16 and the stable structure of NnOMT6–SAH–(*S*)-norcoclaurine was obtained (Fig. 4A). The RMSD of the NnOMT6 skeleton was <6.5 Å, whereas that of (*S*)-norcoclaurine was <2.1 Å (Fig. 4B). Three residues—Asp269, Glu328, and Asn130—in NnOMT6 interacted with (*S*)-norcoclaurine via hydrogen bonding. Moreover, the side chain of Asp269 was in interaction with 6-OH and 7-OH moieties. The 4′-hydroxyl group formed a hydrogen bond with Glu328. The residue Asn130 interacted with the N atom group of (*S*)-norcoclaurine via hydrogen bonding. Eight residues—His165, Asn172, Asn176, Gly207, Asp230, Asp250, Met251, and Lys264—in NnOMT6 formed hydrogen bonds with SAH (Fig. 4C, D). Of these residues, Gly207, Asp230, Asp250, and Lys264 were from motifs I–IV, respectively, which was a highly conserved characteristic of the SAM binding site of plant SAM-dependent methyltransferases [35]. Root mean square fluctuation (RMSF) showed that the four conserved motifs (motif I, D_205_VGGG_209_; motif II, C_226_INFDLPHV_234_; motif III, G_249_DMFV_253_; motif IV, K_264_WICHDW_270_) were stable in the protein (Supplementary Data Fig. S10).

To further identify the key active residues of NnOMT6, the binding energy (∆G_total_) of (*S*)-norcoclaurine with the NnOMT6–SAH complex was estimated using the molecular mechanics/Poisson–Boltzmann surface area (MM/PBSA) approach (Supplementary Data Table S2). Electrostatic interactions dominated NnOMT6–SAH–(S)-norcoclaurine binding, with *∆E*_elec_ being the most beneficial contributor. *∆G*_polar_ was unfavorable for the binding, whereas *∆G*_nonpolar_ was beneficial, resulting in a favorable binding energy overall. The binding free energy of (*S*)-norcoclaurine with the complex was −41.1057 kcal/mol in an aqueous environment. Furthermore, the residues Asn130, Asn323, Asp269, Asp316, Glu328, Leu315, and Leu135 contributed the most to binding free energy by energy composition analysis (Supplementary Data Table S3).

### Site-directed mutagenesis of NnOMT6

A mutant library was constructed for the key amino acids (N130, L135, H165, N176, N323, D316, L315, D269, and E328) of NnOMT6. The catalytic activity of mutants was screened using crude proteins, with six compounds (**1**, **2**, **3**, **7**, **8**, **16**) as substrates (Fig. 4E). The mutant D316A showed the most significant effect, resulting in protein inactivity with all substrates; this finding suggested that D316 plays a key role in catalysis. Compared with wild-type NnOMT6, the mutants N130A, L135A, L135E, L135F, N176A, N176D, N176K, D269A, and E328A showed remarkably decreased catalytic activities for BIA substrates. Because of the interaction of the residues Asn130, Asp269, and Glu328 with the substrate, the alterations observed in them can possibly decrease the binding stability between the proteins and substrates, resulting in decreased activity. The close proximity of L135 and D316 to (*S*)-norcoclaurine (**1**) suggests that they can alter the shape of substrate-binding pockets, thereby influencing the activity. Remarkably, compared with wild-type NnOMT6, the catalytic efficiency of N323A with substrates **2**, **3**, **7**, and **8** was substantially increased. Structural analysis revealed that the side chain of Asn323 for NnOMT6 was extremely close to the 7-OH and 6-OH moieties of (*S*)-norcoclaurine (**1**) on the spatial structure, thereby hindering the acceptance of the methyl residue. By contrast, the short side chain of alanine in N323A could favorably accept a methyl residue (Fig. 4D).

### Probing the catalytic activity of N323A for BIAs

**Figure 5:**
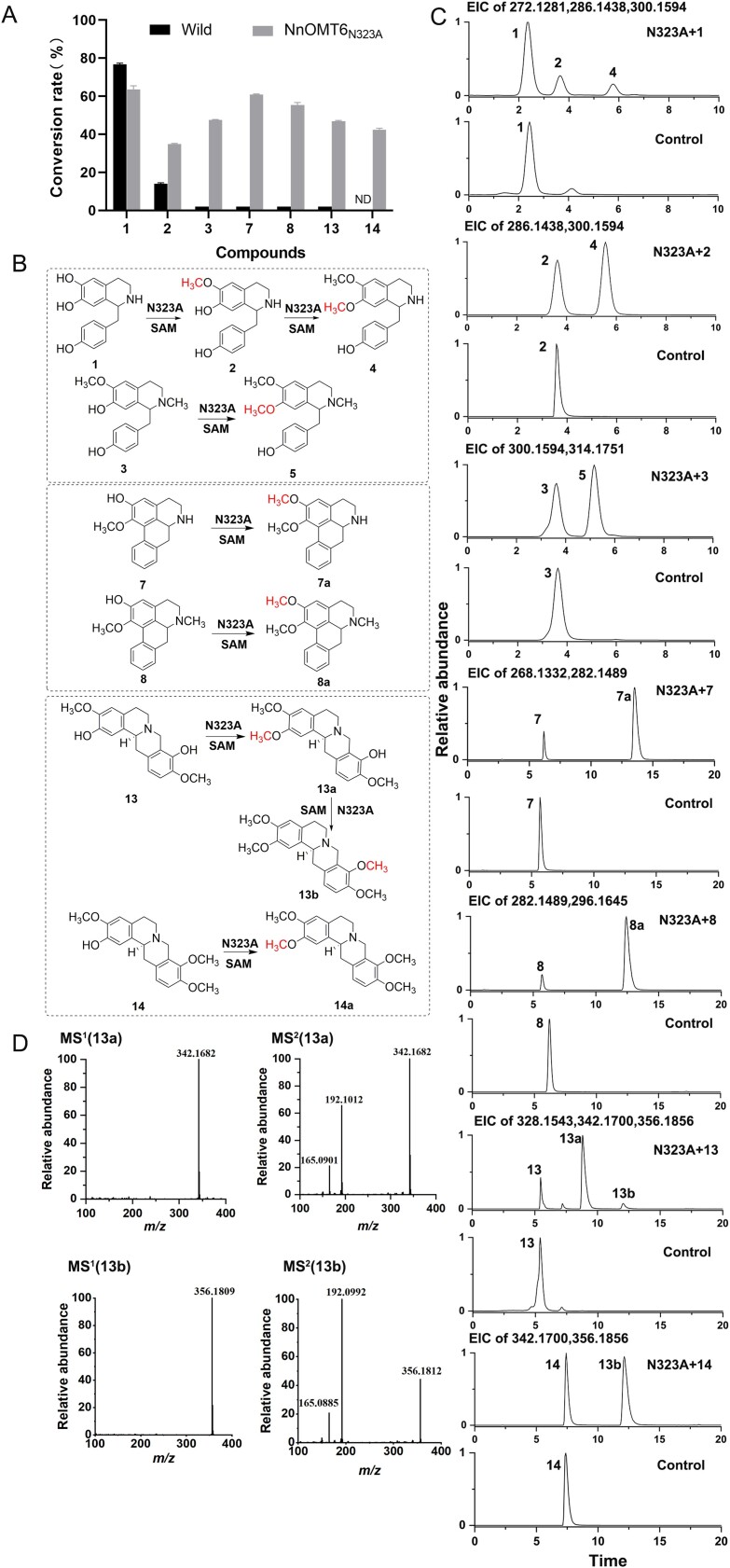
Function characterization of the mutant N323A. (A) *O*-Methylation conversion rates of wild-type NnOMT6 and mutant N323A. (B) The catalytic reaction catalyzed by mutant N323A. (C) Extracted ion chromatogram (EICs) of substrates and products for the enzymatic reactions with compounds **1**, **2**, **3**, **7**, **8**, **13**, and **14** as substrates. (D) Tandem mass spectra for products **13a** and **13b**.

To further investigate the catalytic properties of N323A, purified proteins of the mutant N323A were obtained to test activity using compounds **1**, **2**, **3**, **7**, **8**, **13**, and **14** as substrates (Fig. 5, Supplementary Data Fig. S11). Mutant N323A exhibited an apparently continuous two-step *O*-methylation at the C6 site of compound **1** and the C7 site of compound **2**. Compared with wild-type NnOMT6, the conversion rates for (*S*)-coclaurine (**2**) were increased by ~2.5 times. Interestingly, N323A showed a remarkable increase in catalytic activity toward **3**, **7**, **8**, and **13**, with conversion rates of 47.6%, 61.8%, 55.3%, and 46.8%, respectively. By contrast, NnOMT6 showed weak activity of <2% (Fig. 5A). Furthermore, N323A catalyzed a continuous two-step *O*-methylation at the 2-OH and 9-OH positions of **13**, thereby producing a deprotonated molecule [M + H]^+^ at *m*/*z* 342.1700 (**13a**) and *m*/*z* 356.1856 [M + H]^+^ (**13b**) (Fig. 5B, C). Compound **13b** was identified as (*S*)-tetrahydropalmatine by comparing it with a reference standard (Fig. 5D). Furthermore, it was found that N323A can catalyze the 2-*O*-methylation of **15** to yield (*S*)-tetrahydropalmatine (**13b**), with a conversion rate of 42.4%. These results suggested that N323A exhibited a broader acceptor scope for *O*-methylation and stronger catalytic activity.

The kinetic parameters of N323A were calculated using **7** and **13** (Supplementary Data Fig. S12). The *K*_m_ values of N323A toward asimilobine (132.77 μM) and (*S*)-scoulerine (86.35 μM) were lower than that of NnOMT6 toward (*S*)-norcoclaurine. The *k*_cat_/*K*_m_ values of N323A toward asimilobine and (*S*)-scoulerine were 24.37 ± 0.59 and 20.01 ± 0.99 M^−1^ s^−1^, respectively, which were significantly higher than that of NnOMT6 toward (*S*)-norcoclaurine (16.59 ± 1.48 M^−1^ s^−1^). These results indicated that the catalytic efficiency of mutant N323A to BIAs was relatively higher than that of the wild-type NnOMT6.

## Discussion

To date, ~50 OMTs contributing to BIA biosynthesis have been functionally characterized from different plant species in the order Ranunculales, Magnoliales, and Proteales. These OMTs play a major role in the biosynthesis of structurally diverse BIAs. Although BIAs accumulate as primarily active metabolites in *N. nucifera*, only two OMTs involved in the 1-BIA upstream biosynthesis pathway in *N. nucifera* were identified *in vitro*. In this study, a novel and regiospecific OMT, NnOMT6, was identified from *N. nucifera*; it catalyzes the methylation of phenylpropanoid (caffeic acid and chlorogenic acid), 1-benzylisoquinoline ((*S*)-norcoclaurine, (*S*)-coclaurine, and *N*-methylcoclaurine), aporphine (asimilobine and *N*-methlyasimilobine), and protoberberine (scoulerine) skeletons. NnOMT6 shared a high sequence identity of >70% with three known OMTs (TtOMT, Tt6OMT1, and Tt6OMT2) from *T. tuberosum* and formed a cluster with them in a phylogenetic tree. Therefore, NnOMT6 was determined to be functionally similar to these OMTs and to modify phenylpropanoid and several other BIA skeletons. NnOMT6 can catalyze the 3-*O*-methylation of caffeic acid with the highest catalytic activity. NnOMT6 showed relatively high methylation activity at position 6 of (*S*)-norcoclaurine (76.7%) and weak activity at position 7 of (*S*)-coclaurine (13%) and *N*-methylcoclaurine (<2%) of the 1-BIA skeleton. Interestingly, the methylation function on aporphine backbone without any substituent at C4′ and C3′ positions from *N. nucifera* is the first reported, which was considered to result from *O*-demethylase (ODM) in a previous study [36]. Very low activity (<2%) was observed at the C6 site of the aporphine alkaloids asimilobine, *N*-methlyasimilobine and at C2 position of the protoberberine alkaloid (*S*)-scoulerine (Fig. 3). It was found that the expression pattern of NnOMT6 was highly consistent with the accumulation of coclaurine in different plant parts, suggesting that NnOMT6 is involved in coclaurine biosynthesis in *N. nucifera*.

To further elucidate the catalytic mechanisms of NnOMT6, key residues that chiefly contributed to the activity were obtained by molecular docking and MD simulation using the MM/PBSA method. The role of these residues was verified using site-directed mutagenesis. The residues N130, D269, E328, L135, and D316 proximal to (*S*)-norcoclaurine were mutated, resulting in a large decrease in activity for BIA substrates, especially the mutation of residue D316 causing inactivation of NnOMT6 for all substrates(Fig. 4). These results indicated that these residues had a significant impact on the catalytic activity of NnOMT6, possibly affecting the stability of binding substrates or the distance between donor SAM and acceptors in stereo space. Interestingly, the activity of the mutant N323A for *N*-methylcoclaurine, asimilobine, *N*-methlyasimilobine, and (*S*)-scoulerine substantially increased from <2% to >45%. Moreover, compared with methylation by wild-type NnOMT6, N323A strengthened the 7-*O*-methylation of (*S*)-coclaurine by ~2.5 times. It also exhibited two sequential methylations of (*S*)-norcoclaurine at the C6 and C7 positions. Furthermore, a novel function of N323A was determined—it sequentially catalyzed the methylation of (*S*)-scoulerine at 2-OH and 9-OH, whereas wild-type NnOMT6 only exhibited weak 2- *O*-methylation activity for (*S*)-scoulerine. N323A was found to catalyze (*S*)-tetrahydrocolumbamine, a new acceptor, at the C2 site, with a conversion rate of 42.4% (Fig. 5). These results indicated that N323A can be used for the methylation of phenylpropanoid, 1-benzylisoquinoline, aporphine, and protoberberine skeletons as a biocatalyst in synthetic biology platforms.

In summary, we identified a novel OMT, designated NnOMT6, from *N. nucifera* and determined its regiopromiscuity. NnOMT6 catalyzes the methylation of phenylpropanoid, 1-benzylisoquinoline, aporphine, and protoberberine skeletons. NnOMT6 could catalyze the methylation of caffeic acid at 3-OH, norcoclaurine at 6-OH, coclaurine at 7-OH, *N*-methylcoclaurine at 7-OH, asimilobine and *N*-methlyasimilobine at 6-OH, and scoulerine at 2-OH. Furthermore, we probed and engineered the key residues for *O*-methylation activity using molecular docking and MD simulation, and a mutant library was created. The mutant N323A exhibited higher catalytic efficiency and wider substrate acceptor scope. Findings of the present study revealed efficient biocatalysts for *O*-methylation and provided insights into catalytic mechanisms and protein engineering to strengthen OMT activity in plants.

## Materials and methods

### Plant materials and chemicals

Different tissues of the *N. nucifera* cultivar ‘Hongtailian’ were collected and immediately frozen in liquid nitrogen and stored at −80°C until use. Standards (*S*)-norcoclaurine (**1**), asimilobine (**7**), and *N*-methlyasimilobine (**8**) were purchased from ChemFaces (Wuhan, China); (*S*)-coclaurine (**2**), lirinidine (**9**), liensinine (**10**), and isoliensinine (**11**) were purchased from Chengdu Push Biotech (Chengdu, China); *N*-methylcoclaurine (**3**) and (*S*)-norarmepavine (**4**) were purchased from BioBioPha (Yunnan, China); and armepavine (**5**), lotusine (**6**), N-nornuciferine (**7a**), nuciferine (**8a**), neferine (**12**), (*S*)-scoulerine (**13**), (*S*)-tetrahydropalmatine (**13b**), (*S*)-tetrahydrocolumbamine (**14**), jatrorrhizine (**15**), caffeic acid (**16**), ferulic acid (**17**), chlorogenic acid (**18**), *o*-coumaric acid (**19**), and *p*-coumaric acid (**20**) were supplied from Shanghai Yuanye Biotech (Shanghai, China). SAM was from Sigma–Aldrich (St Louis, MO, USA). BeyoGold™ His-tag Purification Resin was purchased from Beyotime Biotech (Shanghai, China). Acetonitrile, methanol, and formic acid used in UPLC–MS/MS analysis were obtained from Sigma–Aldrich.

### Screening of NnOMT candidate genes

A total of 28 OMTs were selected as query sequences for scanning the transcriptome data of *N. nucifera* by BLASTP searching. The GenBank accession numbers of the 28 OMT sequences were from the literature and are as follows: Ps6OMT (AAP45315.1), PsSOMT3 (AWJ64118.1), PsN7OMT (ACN88562.1), PsOMT2 (AKO60153.1), Ps4′OMT2 (AAP45314.1), PsOMT1 (AKO60152.1), PsSOMT2 (AWJ64116.1), Ps7OMT (AAQ01668.1), PsOMT6 (AKO60157.1), PsSOMT1 (AFK73709.1), and PsOMT7 (AKO60158.1) from *Papaver somniferum*; Tf6OMT (AAU20765.1) from *Thalictrum flavum*; Cc6OMT1 (AXC09385.1) and Cc6OMT2 (AXC09386.1) from *Coptis chinensis*; Cj6OMT (BAB08004.1), Cj4′OMT (BAB08005.1), CjCoOMT (BAC22084.1), and CjSOMT (BAA06192.1) from *Coptis japonica*; NnOMT1 (XP_010244054.1) and NnOMT5 (XP_010276063.1) from *N. nucifera*; Ec4′OMT (BAM37633.1), Ec7OMT (BAE79723.1), and EcSOMT (BBA20643.1) from *Eschscholzia californica*; Ct7OMT (AXC09387.1) and CtSOMT (AXC09384.1) from *Coptis teeta*; and Tt6OMT1(AAD29841.1), TtOMT (AAD29845.1), and Tt6OMT2 (AAD29842.1) from *Thalictrumtuberosum*.

### *De novo* transcriptome assembly and sequencing

In total, 12 fresh lotus tissues, including LS, FS, NR, LBP, RF, LSH, L, P, PT, FSL, O, PS, and SM, were individually ground into powder in liquid nitrogen. Total RNA of 12 lotus tissues was extracted using an RNAprep Pure Kit (Tiangen Biotech, Beijing, China). RNA purity was verified using a NanoPhotometer spectrophotometer (Implen, CA, USA). RNA integrity was assessed using an RNA Nano 6000 Assay Kit on an Agilent Bioanalyzer 2100 system (Agilent Technologies, CA, USA). Thereafter, Illumina paired-end sequencing technology was used.

### Expression patterns of NnOMTs and alkaloid accumulation in different plant parts

Total RNA was extracted from different lotus tissues, including TL, ML, F, and E, using an RNA Easy Fast Plant Tissue Kit (Tiangen Biotech, Beijing, China) and was reverse-transcribed to cDNA using a FastKing RT Kit with gDNase (Tiangen Biotech, Beijing, China). Quantitative RT–PCR was performed using Taq Pro Universal SYBR qPCR Master Mix (Vazyme Biotech, Nanjing, China) on a Cobas Z 480 real-time PCR system (Roche Molecular Systems, Germany). The expression level of NnOMT genes was evaluated by the 2^−ΔΔCt^ method using *β*-actin as the reference with three replicates. Primer information on NnOMTs for qRT–PCR is given in Supplementary Data Table S4.

To extract alkaloids, 100 mg of TL, ML, F, and E fine powder samples were carefully weighed and extracted in 1 ml of 70% methanol and 30% water (v:v) combination, shaken for ~30 seconds at 30-minute intervals six times, and incubated at 4°C overnight. Then, the samples were centrifuged (12 000 rpm, 10 minutes) and the supernatants were filtered with 0.22-μm filters for testing.

### UPLC–ESI–QToF–MS/MS conditions

Determining the content of BIAs and enzyme assay products was performed as described previously [37]. An Agilent 1290 photodiode array and a 6540 triple quad mass time-of-flight mass spectrometer were used for the UPLC–QTOF–MS/MS analysis using a dual electrospray ionization (ESI) detector. A Waters Acquity UPLC CSH C18 Column (2.1 mm × 100 mm, 1.7 μm) was used to separate and analyze the samples with a mobile phase of 0.1% formic acid (eluent A) and acetonitrile (eluent B). The following elution gradient was used: 2% B at 0 minutes, 5% B at 1 minute, 9% B at 5 minutes, 10% B at 12 minutes, 15% B at 16 minutes, 45% B at 20 minutes, 100% B at 22 minutes; flow rate, 0.3 ml/minute; injection volume, 2 μl; and temperature, 35°C.

The ESI source was set to positive ionization mode and the following parameters were set for the QTOF–MS detector: nebulizer, 45 psig; nozzle voltage, 500 V; Vcap, 4000 V; cone voltage, 20 V; sheath gas temp, 350°C; drying gas flow, 8 l min^−1^; sheath gas flow, 11 l min^−1^; collision energy, 30 eV and scan range, *m*/*z* 100–1500 Da. The measured masses were modified by internal references (purine and HP-0921) in real time.

### Molecular cloning and phylogenetic analysis of NnOMT candidate genes

The abovementioned methods were used to extract the total RNAs from frozen leaves and synthesize first-strand cDNAs. NnOMT genes were amplified from cDNA using KOD-plus-neo polymerase (Toyobo) with primers listed in Supplementary Data Table S5 and inserted into a pET-28a expression vector using a ClonExpress^®^ II One Step Cloning Kit (Vazyme Biotech, Nanjing, China).

Alignment of amino acid sequences was performed by ClustalW, and the result was used to construct the phylogenetic tree for NnOMTs and 28 other identified OMTs, using MEGA 7.0 software with 1000 bootstrap replicates.

### Expression and enzyme activity assays of recombinant NnOMTs and mutants

Recombinant plasmids with verified sequences were transformed into *E. coli* BL21 (DE3) (TransGen Biotech, China) for protein expression. The cells were cultured in 300 ml of LB medium (50 μg/ml kanamycin) at 37°C and shaken at 200 rpm until OD_600_ reached 0.6–0.8. Then, IPTG was added to the cultures to a final concentration of 0.3 mM IPTG and induction was performed at 16°C for 20 hours with shaking (180 rpm). The cell pellets were harvested at 4°C by centrifugation and then resuspended with lysis buffer (50 mM NaH_2_PO_4_, 300 mM NaCl, 20 mM imidazole, 2% glycerol, pH 8.0). Thereafter, the suspended cells were disrupted by sonication for 30 minutes in an ice bath, and centrifuged at 10 000 rpm for 40 minutes at 4°C. The supernatant (crude protein) was subsequently used to perform the initial enzyme assays.

The catalytic activity of recombinant NnOMT proteins was initially screened using 13 potential BIA substrates containing monobenzylisoquinolines **1**–**7**, aporphine alkaloids **8**–**10**, and bisbenzylisoquinolines **11**–**13**. The initial reaction was conducted in a 100-μl mixture system comprising 50 mM potassium phosphate (pH 8.0), 200 μM SAM donor, 200 μM acceptor substrate, and 2 mg of crude enzyme. Proteins inactivated by heating at 100°C for 10 minutes were used as negative controls. The mixtures were incubated at 37°C for 12 hours, terminated by the addition of 100 μl methanol, then centrifuged at 12 000 × *g* for 15 minutes at 4°C before UPLC–MS/MS analysis. The activity of mutants was investigated under the same reaction conditions using **1**, **2**, **3**, **4**, **8**, **9**, and **17** as substrates.

### Protein purification and functional characterization of wild-type NnOMT6 and mutant N323A

The crude protein of NnOMT6 and N323A obtained was added to 2 ml of pre-equilibrated BeyoGold™ His-tag Purification Resin (Beyotime Biotech, China) and incubated at 4°C for 4 hours. Thereafter, the lysis buffer and elution buffers containing 250 mM imidazole were used to wash and elute the protein, respectively. The purity of recombinant protein was examined by SDS–PAGE analysis (10% gel) and concentrated using a 10-kDa ultrafiltration tube (Merck Millipore). The concentration of purified enzyme was determined with Bradford Protein Assay kit (TransGen Biotech, Beijing, China).

The catalytic activity of purified NnOMT6 and the mutant N323A was further screened using **1**–**21** as substrates. One hundred microliters of reaction mixture, including 50 mM potassium phosphate buffer (pH 8.0), 200 μM SAM methyl donor, 100 μM substrates, and 50 μg purified protein, was incubated at 37°C for 12 hours. The reactions were analyzed using UPLC–ESI–QTOF–MS/MS.

### Biochemical characterization of NnOMT6 and N323A

The reaction time, pH and temperature of NnOMT6 were optimized using norcoclaurine (**1**) as substrate. Enzyme assays were performed in 50 mM potassium phosphate buffer (pH 5.0–8.5) and 100 mM Gly–NaOH buffer (pH 9.0–11.0) at 37°C for 4 hours to obtain the optimal pH. The reactions were conducted at 4–60°C for 4 hours (pH 8.0) to optimize temperature. The linear range of product formation was determined for 5–360 minutes.

The kinetic parameters of NnOMT6 were determined in a 100-μl reaction mixture containing 50 mM potassium phosphate (pH 8.0) and varying concentrations of **1** (4–500 μM) or **17** (20–500 μM) at a fixed concentration of 200 μM SAM or varying concentrations of SAM (4–500 μM) at a fixed concentration of 200 μM **17**. For the kinetic studies of the mutant N323A, reactions were performed with **8** or **14** at a concentration of 4–400 μM in a total volume of 100 μl. The reactions were incubated at 37°C for 30 min and terminated by adding 100 μl methanol. All reactions were conducted in triplicate. The kinetic data were calculated by non-linear regression analysis using a Michaelis–Menten model in GraphPad Prism 8.0 software.

### Homology modeling, molecular docking, and molecular dynamics simulation

The structural model of NnOMT6 was constructed using SWISS-MODEL [38, 39], The crystal structure of COMT (PDB ID: 1KYW) was used as the template in homology modeling. Molecular docking of NnOMT6 with norcoclaurine was conducted using MOE Dock software, version 2015.10 (Chemical Computing Group, Montreal, Canada) [40].

After docking, the complex NnOMT6–SAH–norcoclaurine was optimized using MD simulation performed with AMBER16 [41]. To neutralize protein structure, sodium/chlorine counterions were added. Solvation of each protein structure was conducted in a cuboid box of TIP3P water molecules with 10-Å solvent layers between the edges of the box and the surface of the solute. The protein was subjected to AMBER GAFF and FF14SB force fields. All covalent bonds involving hydrogen atoms were restricted using the SHAKE algorithm with a time step of 2 FS. Long-range electrostatic interactions were treated by the particle mesh Ewald method. For each solvated system two steps of minimization were performed before the heating step. The initial minimization was conducted with 4000 cycles with all heavy atoms restrained using 50 kcal/(mol Å^2^), whereas solvent molecules and hydrogen atoms were free to move. Then, the stage of non-restrained minimization was performed with the steepest descent minimization for 2000 cycles and conjugated gradient minimization for 2000 cycles. Thereafter, the entire system was heated from 0 to 300 K in 100 ps using Langevin dynamics at a constant volume, following which it was equilibrated for 150 ps at a constant pressure of 1 atm. Periodic boundary dynamics simulations were performed for the entire system in an NpT ensemble (constant composition, pressure, and temperature) at a constant pressure of 1 atm and 300 K during the production step. In the production phase, 100-ns simulation was conducted. The binding free energy of the complex was calculated using the MM/PBSA method.

### Site-directed mutagenesis of NnOMT6

The mutant plasmids of NnOMT6 were cloned with pET28a-NnOMT6 vector as the template using a Fast MultiSite Mutagenesis System kit (TransGen, China). Primer pairs used for PCR amplification are shown in Supplementary Data Table S6.

## Supplementary Material

Web_Material_uhac276

## Data Availability

All data supporting this research can be obtained in the paper and within its supplementary materials published online.
